# Pulmonary hydatidosis genotypes isolates from human clinical surgery based on sequencing of mitochondrial genes in Fars, Iran

**DOI:** 10.1186/s13019-021-01547-2

**Published:** 2021-06-07

**Authors:** Parviz Mardani, Ali Talebi Ezabadi, Bahareh Sedaghat, Seyed Mahmoud Sadjjadi

**Affiliations:** 1 Department of Surgery, Shiraz University of Medical Sciences, Shiraz, Iran; 2 Thoracic and Vascular Surgery Research Center, Shiraz University of Medical Sciences, Shiraz, Iran; 3 Department of Parasitology and Mycology, School of Medicine, Shiraz University of Medical Sciences, Shiraz, Iran

**Keywords:** Hydatid cyst, Genotype, Iran, Pulmonary

## Abstract

**Background:**

Cystic echinococcosis (CE)/hydatidosis is an important neglected parasitic zoonotic disease caused by the metacestode of *Echinococcus granulosus* s.l. The present study was designed to identify the pulmonary CE species/genotypes in isolated human underwent to surgery in our center in Southern Iran.

**Methods:**

The study population of this study were all patients in Fars province who were admitted to Namazi Hospitals for pulmonary hydatid cyst surgery. Thoracic surgery was performed in the thoracic ward and the cyst/s was removed by open surgery via posterolateral or lateral thoracotomy. DNA was extracted from the germinal layer or the protoscoleces. PCR technique was performed using the cytochrome C oxidase subunit1 (cox1) gene, and the products were sequenced.

**Results:**

A total of 32 pulmonary hydatid cyst samples were collected from 9 (28%) female and 23 (72%) male aged from 4 to 74 years old. A total of 18(56%) cyst/s were in the left lobe and 14 (44%) cysts in the right lobe. Sequence analysis of the cysts showed that 24 samples (75%) were *E. granulosus* s.s (G1-G3) genotype and 8 (25%) were *E. canadensis* (G6/G7) genotype.

**Conclusion:**

*E.granulosus* s.s genotype was the most prevalent genotype followed by *E. canadensis* (G6/G7) genotype. There was no significant statistical correlation between cysts’ size, location, genotype strain, and patients’ age and gender.

## Background

Cystic Echinococcosis (CE), also known as hydatid disease or hydatidosis, is a neglected zoonotic disease. Canids are the definitive hosts of the parasite and various ungulates as its intermediate hosts [1]. Human infection is caused by the ingestion of the eggs of *Echinococcus granulosus* s.l. leading to the formation of larval stage in the human body [1]. The liver is the most commonly affected organ (about 70% of cases) and the lungs are the second most commonly affected organ (about 20% of cases). In 85–90% of CE cases, only one organ is affected, and greater than 70% of cases have only one cyst [2, 3]. There are currently nine species of *Echinococcus*.; four of them have been known to cause public health concern, which includes *Echinococcus granulosus* s.l. causing cystic echinococcosis, *Echinococcus multilocularis* producing alveolar echinococcosis, and *Echinococcus vogeli* and *Echinococcus oligarthrus* resulting in polycystic echinococcosis. Furthermore, two new species have lately been known: *Echinococcus shiquicus* and *Echinococcus felidis* [4–6]. *E. granulosus* sensu lato consist of 5 species of which 4 (*E. granulosus* s.s., *E. equinus*, *E. ortleppi*, and *E. canadensis)* are responsible for human CE (*E. ortleppi* and *E. equinus* to a far lesser extent, but they infected humans after all) with *E. multilocularis, E. oligarthrus,* and *E.vogeli* there are 7 species causing disease in human.

Recent advances in phylogenetic systematics have resulted in the recognition of nine species of *Echinococcus*: *E. granulosus* sensu stricto (G1 to G3), *E. equinus* (G4), *E. ortleppi* (G5), *E. canadensis* (G6 to G10), *E. multilocularis*, *E. vogeli*, *E. oligarthrus*, *E. felidis*, and *E. shiquicus* [5].

The most important etiological agents of human CE are *E. granulosus* s.s. and *E. canadensis*, both of which were formerly included in the species complex of *E. granulosus* s.l [7]. Different species and genotypes including *E. granulosus* s.s, *E. canadensis*, *E. ortleppi* have been reported from humans and animals from Iran [8–10]. CE can present various clinical manifestations depending on the location of involvement [11]. In pulmonary hydatidosis, the lower lobes are more frequently affected than the upper ones, and one in five cases is bilateral. The scale varies from 1 to 20 cm and the lung tissue is the organ where the hydatid cyst will become the largest due to simple compressibility. Lung cysts will stay asymptomatic and thus quiet for years, emerging for imaging or other purposes until chest X-rays are required. Coughing, discomfort, chest pain, fever, and hemoptysis are the initial symptoms. The development of an anaphylactic reaction and the appearance of vomiting are indicative of the rupture of the cyst. In this latter case, the direct study of sputum will show the presence of *E. granulosus* s.l. hooklets. When confronted with an initial thoracic hydatid cyst, it is mandatory to preclude the presence of a liver location [12]. The preliminary growth of the hydatid disease of the lung or lung echinococcosis is asymptomatic and some symptoms, such as chest pain, cough, fever, dyspnea, or allergic reaction, hemoptysis may appear [13, 14].

Human CE is treated by a variety of pharmacological and surgical methods ranging from chemotherapeutic treatment with albendazole and minimally invasive surgical treatments like PAIR (percutaneous Puncture, Aspiration, Injection, and Reaspiration) in asymptomatic patients [15, 16]. There are various surgical procedures for pulmonary hydatid disease like enucleation, pericystectomy, cystostomy with capitonnage, cystostomy with the closure of the bronchial openings, and capitonnage, cystotomy with the closure of bronchial openings alone, open aspiration by Figuera technique, segmental resection, and lobectomy [17, 18]. Currently, the most accepted surgical procedure for lung pulmonary is cystostomy methods that including intact cyst enucleation or removal after needle aspiration by maintaining the lung parenchyma as much as possible [14, 19–21].

CE is most commonly associated with the liver [22]. On the other hand, certain echinococcus species appear to have a predilection for other organ systems. This has been shown by the studies in Iran and Mongolia where the cases of brains have been reported to be caused primarily by *E. canadensis*(G6) [23, 24]. This may indicate that certain *E. canadensis* variants have brain or liver predilections [24]. These studies show that the molecular identification of the causative species is essential.

Different genotypes of *E. granulosus* s.l. have been identified from different organs based on molecular works in the world [4, 7, 23]. Numerous molecular studies on human isolates of *E. granulosus* s.l. have recognized the genotypes G1, G3, and G6 in different regions of Iran [9, 23, 25–27].

Iran remains one of the endemic countries for hydatid disease [28] and CE is responsible for 1% of surgery ward admissions in Iran [29]. Genotyping of human hydatid cyst is useful because it provides data about the transmission patterns of the *Echinococcus sp*. and information on the predominant prevalence of special genotypes in humans. It can also provide valuable information in the design of human hydatidosis control strategies [29, 30].

On the other hand, the most prevalent genotypes of human CE in Iran have been reported to be G1 and G6 [9, 31, 32]. This study was aimed to evaluate genotypes diversity of isolated human lung hydatid cysts based on mitochondrial genetic sequencing and find out the most prevalent species/genotype of pulmonary hydatidosis in Fars Province, Southern Iran.

## Methods

### Sample collection

**Fig. 1:**
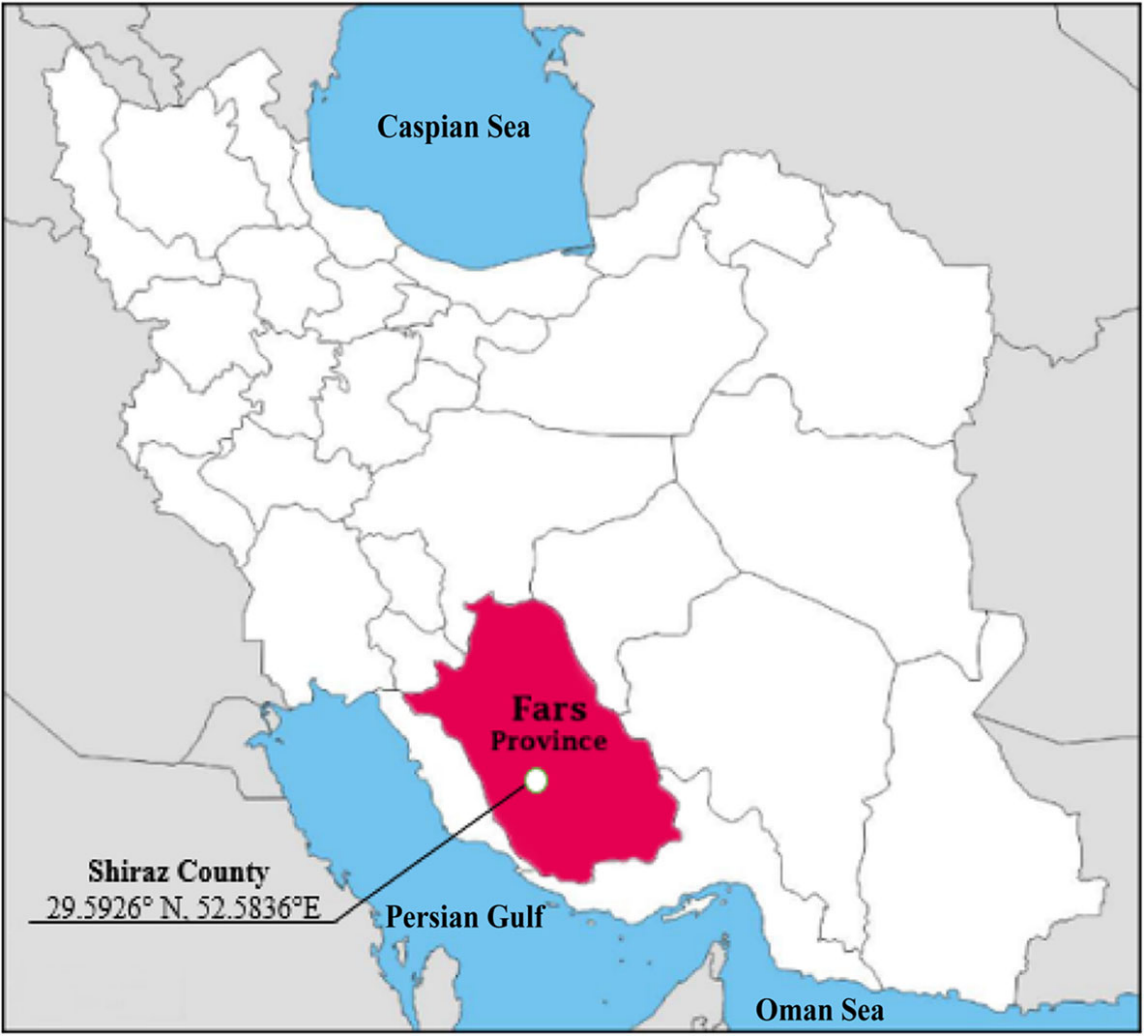
Iran map showing the situation of Fars province

**Fig. 2:**
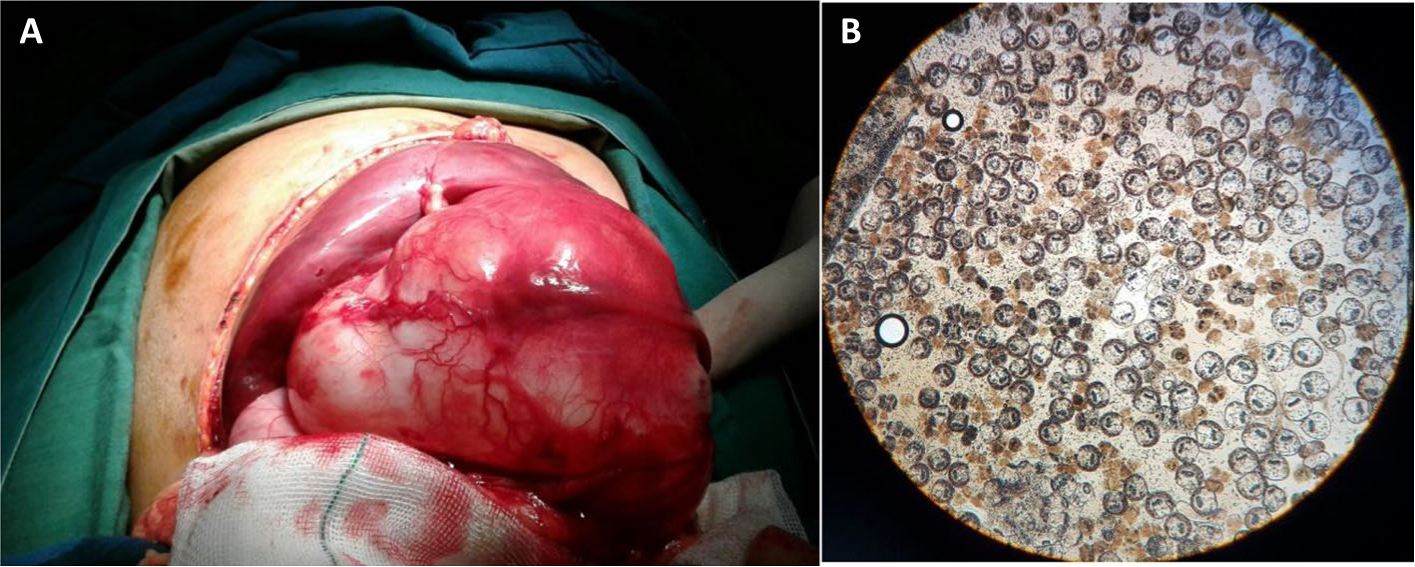
**A:** Surgery of pulmonary hydatid cyst **B:** Protoscoleces of pulmonary hydatid cyst

**Fig. 3:**
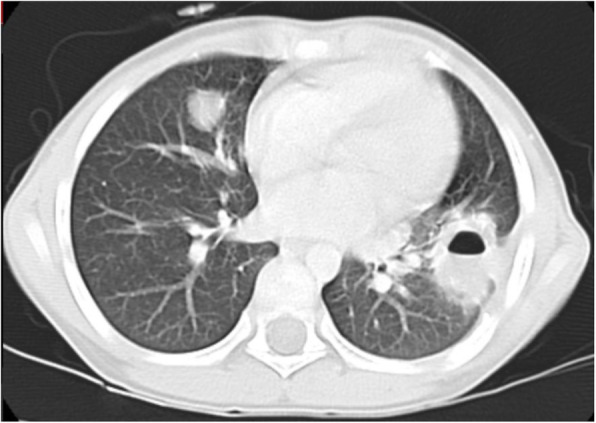
computed tomography of pulmonary hydatid cyst in in base of left lower lobe

A total of 32 patients with lung CE from different counties of Fars province, southern Iran (Fig. 1), who had been referred to different hospitals affiliated to Shiraz University of Medical Sciences, Shiraz, Iran were operated on during 2017–2019 (Figs. 2 and 3). Clinical and demographical features of the patients were collected, using the patients’ hospital records. Lungs cysts were collected and transferred to the Helminthology Research Laboratory at the School of Medicine, SUMS, Iran. A part of the cysts was sent to the pathology section for CE confirmation. This study was approved by the Ethics Committee of Shiraz University of Medical Sciences (Code: IR.SUMS.MED.REC.1397.531). Informed consent was obtained from all individual participants included in the study. Patients were informed of the study objectives and gave written informed consent for tissue samples to be used for research.

### Surgical procedures

The applied surgical procedure was open surgery via posterolateral or lateral thoracotomy. The method of surgery was performed based on cyst properties. In patients with an intact cyst, after wrapping the pleural cavity with lap sponge soaked with hypertonic saline, the suction of the cyst fluid was carried out with an angiocatheter, and subsequently enucleation of the laminated layer of the cyst, followed by closing the bronchial opening by vicryl suture. Also, capitonnage was performed accordingly.

In cases of ruptured cysts and the involvement of pleural cavity and peel formation, decortication of the cyst was performed followed by enucleation or wedge resection. Lobectomy was performed in cases in which consolidation of the lung parenchyma was observed. In cases in which the cyst was located in the periphery of the lung parenchyma, wedge resection was performed without any manipulation of the cyst. Furthermore, in patients with bilateral cysts, the operation was carried out in two stages with a 4-week interval, and the first stage was performed on the larger and intact cyst. Chest tube was inserted in all patients accordingly, which was removed after 4 to 5 days.

### DNA extraction and PCR amplification

DNA was extracted from the germinal layers of the cyst/s using a commercial DNA extraction kit (Yekta Tajhiz Azma, Iran), PCR, targeting *cox1* genes, which was performed according to previous work procedures [33].

A 446 bp of the partial region of the cox1 gene was amplified for each extracted DNA.

The Cytochrome c oxidase subunit 1 (*cox1*) gene was amplified by two primers as follows: JB3 (5′-TTTTTTGGGCATCCTGAGGTTTAT-3′) and JB4.5 (5′-TAAAGAAAGAACATAATGAAAATG-3′) for *cox1* gene as forward and reverse primers, respectively (35). PCR was performed in a final volume of 50 μl, including 2.5 μl genomic DNA, 3.5 mM MgCl2, 250 μM of dNTPs, 25 p mol. of each primer and 2 U of Taq polymerase. The temperature profile was used for DNA amplification as follows: 40 cycles of 94 °C for 45 s, 51 °C for 35 s, 72 °C for 45 s, followed by a final extension at 72 °C for 10 min. PCR products were visualized using electrophoresis with 1.5% agarose gel in TAE buffer and stained with GelRed (Biotium®). A 100-bp molecular ladder was used as DNA size marker in each gel for estimating the size of the bands. Gels were observed and photographed using a UV-trans illuminator (Uvitec®).A purification kit (Vivantis®) was used for PCR primary products *cox1* gene purification followed by sequencing in two directions using the similar forward and reverse primers applied in the PCR.

### Sequencing and phylogenetic analysis

PCR products were sequenced by Bioneer Corporation (South Korea). Using BioEdit, the sequenced genes were edited to equal lengths for the phylogenetic analysis and using the BLAST program of GenBank, the obtained sequences were aligned and compared.

A Phylogenetic tree was drawn using our sequences and reference sequence obtained from GenBank. The maximum likelihood tree was constructed based on the Tamura-Nei model, using the MEGA5 software [34]. *Taenia saginata* was considered as, theoutgroup in the model.

## Results

### Demographic characteristics of patients

A total of 9 out of 32 patients (28%) patients were female and 23 out of 32 (72%) were male. Patients’ age ranged from 4 to 78 years and the mean age was 31.8 (SD = 20.28) years (Table 1).

**Table 1:** Distribution of various species/ genotypes of pulmonary hydatid cyst based on patients’ demographic and clinical information

Variable	Echinococcus species / genotypes (G1-G3), *n* (%)	Echinococcus species / genotypes (G6/G7), *n* (%)	Total (%)
Age (years)			
≤ 20	8 (23)	3 (9)	11 (34.5)
21–40	6 (19)	4 (12.5)	10 (31.5)
40–60	8 (25)	0 (0)	8 (25)
> 60	3 (7)	1 (3)	3 (9)
Gender			
Male	18 (56.5)	5 (15.5)	23 (72)
Female	6 (19)	3 (9)	9 (28)
lung lobe			
Left	15 (47)	3 (9)	18 (56)
Right	9 (28)	5 (16)	14 (44)
Size of cyst			
Small	11 (34)	6 (19)	17 (53)
Medium	11 (34.5)	2 (6.5)	13 (41)
Large	2 (6)	0 (0)	2 (6)
Total (%)	24 (75)	8 (25)	32 (100)

### Characteristics of the cysts

According to the location of the pulmonary hydatid cysts: The cyst of a total of 18 cases (56%), was in the left lobe, and in 14 cases (44%) the cysts were in the right lobe. The cysts’ size was classified as small (0–4 cm), medium (5–9 cm), and large (10–15 cm). Among all operated hydatid cysts, 17 (53%) were small, 2 (6%) were large and 13 (41%) of them were medium size (Table 1). Figure 3 demonstrates a radiological image of pulmonary hydatid cyst.

### PCR, nucleotide sequence analysis and phylogenetic analysis

The PCR products showed a specific band of 446 bp. The PCR products were sequenced using the same primer as PCR. Blast analysis showed a total of 24 out of 32 isolates (755%) to be *E. granulosus* s.s.(G1-G3). A total of 8 out of 32 isolates (25%) were *E. canadensis* (G6/G7). The sequenced genes in this study were deposited in the GenBank under the accession numbers from MW350092 to MW350101.

**Fig. 4:**
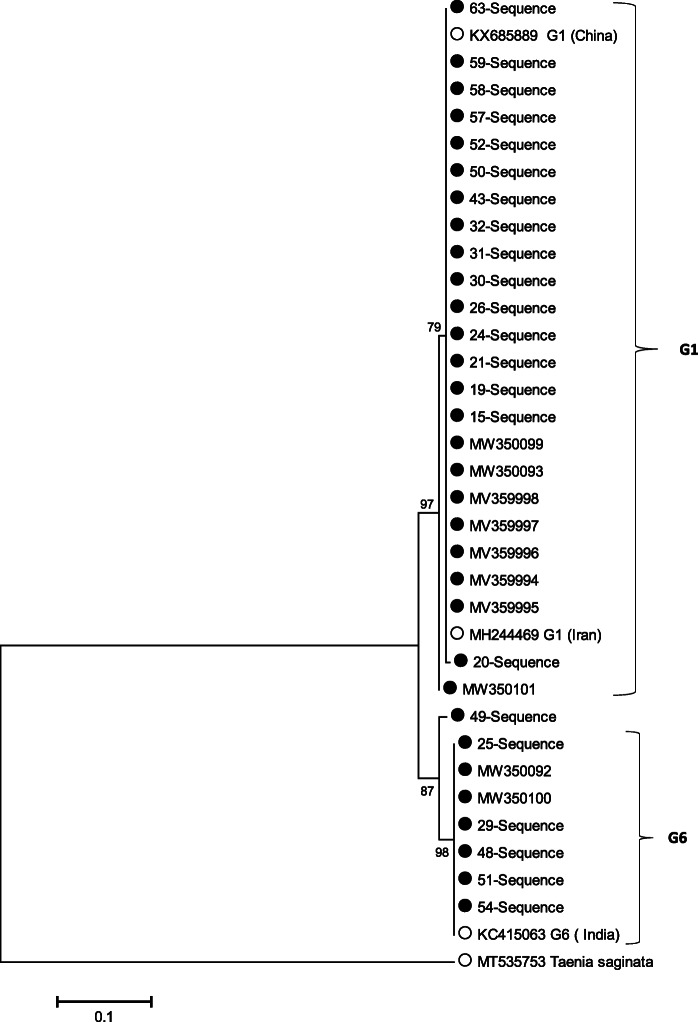
Molecular phylogenetic tree of mitochondrial cox1 region of *E. granulosus* s.s. and *E. canadensis* (G6/G7) isolates from human. Phylogenic analysis was done based on partial cox1 gene sequences data obtained in the present study and other sequence and species and genotypes of *Echinococcus* using maximum likelihood method with MEGA 5 software

The amplified genes and the accession numbers for the detected genotypes and other genotypes used for comparison are shown in Table 2. *Taenia saginata* with GenBank accession number MT535753 and *Echinococcus granulosus* strain G6 cytochrome oxidase subunit I gene with GenBank accession numbers: KC415063, were used as out groups in the phylogenetic trees, respectively. Phylogenetic tree construction showed the relationships between the isolates from the present study and the reference sequences from *E. granulosus* s.l. genotypes and other species (Fig. 4).

**Table 2:** Information about sequences that used for phylogenetic analysis of cox1 genes

Accession number (cox1)	Genotype of *Echinococcus*	Reference	Accession number (cox1)	Genotype of *Echinococcus*	Reference
MW350093	G1	This study	MW350101	G1	This study
MW350094	G1	This study	MW350100	G6	This study
MW350095	G1	This study	MW350092	G6	This study
MW350096	G1	This study	MT535753	*Taenia saginata*	Omondi et al. (2020) [35]
MW350097	G1	This study	KX685889	G1	Shang et al. (2019) [36]
MW350098	G1	This study	KC415063	G6	Sharma et al. (2013) [37]
MW350099	G1	This study	MH244469	G1	Neysi et al. (2020) [25]

Statistical analysis showed no significant correlation between the genotype strains and the patients’ gender, age, and also the involving lung lobe and cyst size. Table 1 shows the diversity and distribution of different strains based on various demographic and clinical features of the patients.

## Discussion

Cystic echinococcosis/hydatidosis, as a zoonotic disease, involves patients worldwide and causes many related economical and public health consequences annually [38]. The disease is also known as a major endemic disease in Iran, where sheep, goats, and cattle, are still slaughtered traditionally and corpses wastes are simply available for stray dogs and other wild animals [39]. Since Namazi Hospital is a referral thoracic surgery center in the south of Iran and a large number of patients in Fars province and surrounding provinces with pulmonary hydatid cysts are admitted there, the present study aimed to evaluate the genotypes of isolated lung cysts, based on the sequencing of mitochondrial genes, in Fars province.

*E.granulosus* s.l.species has an extensive variety in the host and geographical distribution by diversity as high as 10 different well-known genotypes globally [22, 38]. Currently genotypes of *E.granulosus* s.l. are grouped as *E. granulosus* sensu stricto (s.s.) (G1 -G3), *E. equinus* (G4), *E. ortleppi* (G5), *E. canadensis* (G6, G7, and G8, G10) and E. felidis [5, 38, 40]. Among them, *E. canadensis* and *E. ortleppi* are pathogenic to human, and *E. granulosus* s.s. responsible for most of human CE in the world [7]. Recently for the first time, *E. equinus* has recently been reported from humans [41].

Its genotype diversity would implicate pathogenicity, specific hosts, and more importantly, helps designing vaccines and effective medications in different geographical settings [42]*.* Such studies and related factors provide researchers with information about the state of CE transmission in the country and also a basis for the scope and type of subsequent studies.

In the present study, the genotypes of 32 samples were confirmed by sequencing. A total of 24 samples (75%) were *E. granulosus* s.s.(G1-G3) genotype and 8 samples (25%) were *E. canadensis*(G6/G7) genotype.

In the study of genotypes of hydatid cysts, using the cox1 gene sequence, the G1 genotype was identified as the dominant genotype of *E. granulosus* s.l. in human specimens according to other reports from Iran [4, 25, 26, 29]. The G1 genotype has also been reported in animals including sheep, cattle, goats, and wild boars in Iran [29].

With the analysis of the mitochondrial cox1 gene, 8 samples (25%) had the G6/G7 genotype. The presence of *E. canadensis* (G6) genotype (camel genotype) has previously been reported in domestic animals and humans in different countries including Iran [23, 43–45]. In the study of Sadjjadi et al. the G6 genotype was confirmed in the paraffin-embedded tissue of cerebral echinococcal cysts, stating that the G6 genotype has a higher affinity for the human brain than the G1 strain [23].

Our data showed that *E. canadensis* is responsible for 25% of human pulmonary CE cases. The active camel-dog cycle of Echinococcus is present in many parts of the country especially camels and sheep breeding areas. The diversity of these results can be explained by the type of studied samples, the differences in the geographical area, and the method used to determine the genotype.

In the present study, the highest level of infection was seen in the age group of 21 to 30 years, and the lowest infection in the age range of 61 to 70 years. No statistical association was observed between the patient’s age and the parasite strain. This could be due to the low sample size.

According to previous studies, women are more likely to come in contact with sources of infection, such as dogs, dirt, vegetables, such that the prevalence of CE is more in females than males [29]. However, this is not unique everywhere such that in some areas, males have been the highest involved population [15]. It could be due to the culture and social criteria. In our study from the 32 patients with pulmonary hydatid cysts, 9 patients (28%) were female and 23 (72%) were male with a statistically insignificant difference.

In the current study, 18(56%) of the studied patient had pulmonary hydatid cysts in the left lobe and 14 (44%) cases had in the right lobe. No significant association was found between the location of the cyst in the lung and the parasite genotype. Our study showed that small hydatid cyst sizes were observed more than other sizes pulmonary CE causes the symptoms related to lung cysts such as cough, chest pain in the early stages of the disease when cysts are small in size, and patients seek treatment. Hence, the disease in the early stages is often stopped with surgery or medication, and the chances of the cyst enlarging and reaching the medium size and large size are reduced. Statistical analyzes showed no significant association between cyst size and parasite strain. However, it is suggested that similar studies be performed on larger scales to determine the genotype of *Echinococcus* in the province and other areas. Although, it is postulated that some Echinococcus species have preferences for certain organs of their hosts. This can only be investigated if the causative species is identified [23, 24]. The results of the present work do not show such a correlation.

## Conclusion

*E. granulosus* s.s. followed by *E. canadensis*(G6/7) were the most frequent species/genotypes in CE lungs in southern Iran. However, there was no significant statistical association between cysts’ size, location, genotype and, patients’ age and gender.

## Data Availability

All data and materials are available upon request.

## Abbreviations

CE: Cystic echinococcosis
PAIR: Percutaneous Puncture, Aspiration, Injection, and Reaspiration
